# Supplementary material for: Dried Blood Spots as Matrix for Evaluation of Valproate Levels and the Immediate and Delayed Metabolomic Changes Induced by Single Valproate Dose Treatment
Source: Int J Mol Sci. 2022 Jun 25;23(13):7083. doi: 10.3390/ijms23137083 (PMC9266449; doi:10.3390/ijms23137083)

Supplementary Information

Dried blood spots as matrix for evaluation of valproate levels and the immediate and delayed metabolomic changes induced by single valproate dose treatment

Sing Teang Kong[1,+], Hai-Shu Lin[2,1,+], Jianhong Ching[3], Huiqing Xie[4,*] and Paul Chi-Lui Ho[1,*]

[1]Department of Pharmacy, National University of Singapore, 18 Science Drive 4, Singapore 117543.

[2]College of Pharmacy, Shenzhen Technology University, 3002, Lantian Road, Pingshan District, Shenzhen 518118, China

[3]Duke-NUS Medical School, 8 College Road, Singapore 169857.

[4]Institute of Materials Research and Engineering, Agency for Science Technology and Research, Singapore 138634.

Table S1. The top 5 ranking compounds to differentiate the control from the treatment group at the respective time points in the male rats.

Table S2. The respective average responses and their standard deviations (SD) of the 10 metabolites detected in DBS stored in freezer at -20°C for 0, 6, 24 and 48 hr. The last column showed the relative standard deviation (RSD) of the average response in the detection of the respective metabolite over the entire period from 0 to 48 hr.

Table S3. The respective average responses and their standard deviations (SD) of the 10 metabolites detected in DBS stored at room temperature (25°C) for 0, 6, 24 and 48 hr. The last column showed the relative standard deviation (RSD) of the average response in the detection of the respective metabolite over the entire period from 0 to 48 hr.

Table S4. The percentage recoveries (Mean ± SD) from DBS of alanine, palmitic acid and cholesterol spiked at two different concentrations.

Figure S1. Validation plot acquired after 100 times data permutations, no over-fitting of the model was confirmed based on the criteria that all permuted R^2 and Q^2 values on the left are lower than the original point on the right, and the Q^2 regression line has a negative intercept.

Table S1. The top 5 ranking compounds to differentiate the control from the treatment group at the respective time points in the male rats. The non-identifiable compounds were labeled as m/z value @ retention time.

0.5 hr	2.5 hr	5 hr	8 hr	24 hr
69.0@18.231447	**pipecolic acid 2**	57.0@20.116108	57.0@20.116108	57.0@17.10838
69.0@15.317517	69.0@15.346338	**tetratriacontane**	204.0@15.893225	69.0@15.346338
71.0@12.928356	197.0@8.251593	71.0@21.317522	69.0@15.731224	69.0@15.317517
71.0@22.898218	189.0@9.008106	**stearic acid**	71.0@20.970842	73.0@16.7488
**2-hydroxybutyric acid**	84.0@12.152817	69.0@18.121449	**stearic acid**	191.0@7.6123276

Table S2. The respective average responses and their standard deviations (SD) of 10 metabolites detected in DBS stored in freezer at -20°C for 0, 6, 24 and 48 hr. The last column showed the relative standard deviation (RSD) of the average response in the detection of the respective metabolite over the entire period from 0 to 48 hr.

Peak Number	Retention Time (in minutes)	0 hr	6 hr	24 hr	48 hr	RSD (%)
		Average ± SD				
1	6.37	29.20±1.63	27.24±3.72	24.22±0.86	25.53±2.28	5.90
2	6.46	25.12±1.62	25.46±1.75	24.09±1.16	24.54±0.87	2.84
3	7.08	20.18±1.57	21.93±3.47	20.03±0.78	20.66±0.83	4.62
4	7.11	20.29±1.53	20.94±2.36	18.03±0.43	18.65±1.63	8.00
5	7.80	6.22±1.35	6.09±0.92	6.04±0.03	6.11±0.48	0.62
6	8.89	19.78±1.29	21.47±0.90	22.06±0.22	21.31±2.21	1.83

7	9.45	9.02±0.58	8.37±2.20	9.61±0.77	9.63±0.69	7.84
8	16.86	47.26±4.83	40.28±5.74	43.49±3.44	52.79±4.60	14.27
9	18.46	3.62±0.28	4.33±0.52	4.42±0.21	4.27±0.52	1.70
10	27.00	30.83±0.80	41.29±2.12	32.72±7.29	44.70±9.70	15.58

Table S3. The respective average responses and their standard deviations (SD) of the 10 metabolites detected in DBS stored at room temperature (25°C) for 0, 6, 24 and 48 hr. The last column showed the relative standard deviation (RSD) of the average response in the detection of the respective metabolite over the entire period from 0 to 48 hr.

Peak Number	Retention Time (in minutes)	0 hr	6 hr	24 hr	48 hr	RSD (%)
		Average ± SD				
1	6.37	29.20±1.63	24.96±1.46	25.31±1.05	26.42±2.21	5.86
2	6.46	25.12±1.62	22.80±0.95	25.36±0.41	25.77±2.08	4.16
3	7.08	20.18±1.57	19.08±1.06	20.71±0.34	21.65±1.62	5.55
4	7.11	20.29±1.53	17.37±0.80	19.32±0.60	20.34±1.44	4.62
5	7.80	6.22±1.35	5.66±0.54	5.98±0.09	5.87±0.39	9.45
6	8.89	19.78±1.29	22.16±1.32	21.62±0.83	22.45±2.52	5.98
7	9.45	9.02±0.58	9.74±0.23	9.83±0.90	9.82±1.25	2.38
8	16.86	47.26±4.83	53.53±5.26	40.19±5.03	49.16±5.34	9.83
9	18.46	3.62±0.28	3.75±0.31	4.27±0.22	5.08±0.30	3.32
10	27.00	30.83±0.80	31.79±5.11	42.09±12.41	47.31±3.93	16.07

Table S4. The percentage recoveries (Mean ± SD) from DBS of alanine, palmitic acid and cholesterol spiked at two different concentrations.

**Analyte**	**Molecular weight**	**LogP**	**Concentration**	**Mean absolute recoveries ± SD (%)**
Alanine	89.09	-0.68	1 μg/mL	125.32 ± 0.37
			100 μg/mL	88.94 ±1.26
Palmitic acid	256.42	6.26	1 mg/mL	99.45 ± 1.92
			5 mg/mL	98.81 ± 6.15
Cholesterol	386.79	7.11	1 mg/mL	69.77 ±1.84
			2mg/mL	55.58 ±1.96

Figure S1. Validation plot acquired after 100 times data permutations, no over-fitting of the model was confirmed based on the criteria that all permuted R^2 and Q^2 values on the left are lower than the original point on the right, and the Q^2 regression line has a negative intercept.

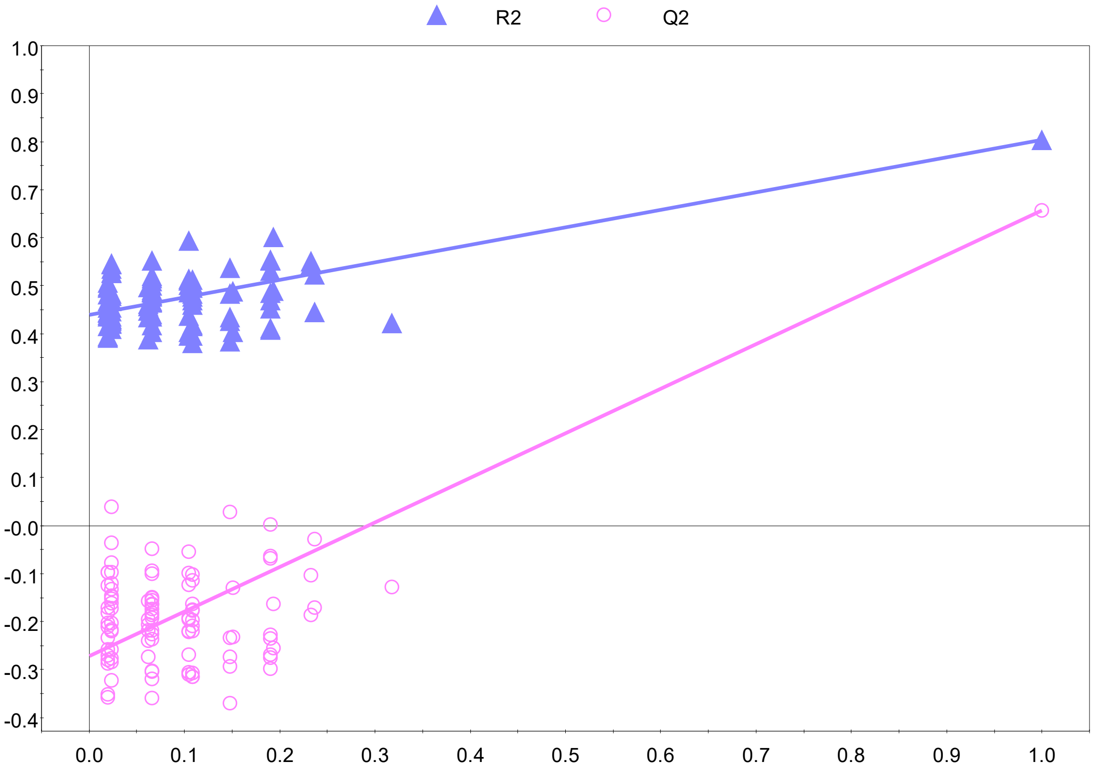

Supplement: Supplementary file 1 [file ijms-23-07083-s001.zip › ijms-1749412-supplementary.pdf]
